# TAPISTRY: A Phase II Study of Atezolizumab in Patients with Tumor Mutational Burden–High Tumors

**DOI:** 10.1158/1078-0432.CCR-25-3336

**Published:** 2026-01-09

**Authors:** David M. Thomas, Jeong Eun Kim, Fabrice Barlesi, Uwe M. Martens, Maciej Krzakowski, Rafal Dziadziuszko, Jae Ho Jeong, Gennaro Daniele, Timothy R. Wilson, Felice Wu, Brian P. Simmons, Sid Patel, Maria Sbirnac, Monika Kaul, Shirish M. Gadgeel

**Affiliations:** 1 Centre for Molecular Oncology, University of New South Wales, Sydney, Australia.; 2 Department of Oncology, Asan Medical Centre, University of Ulsan College of Medicine, Seoul, Republic of Korea.; 3 Department of Medical Oncology, International Centre for Thoracic Cancers (CICT), Gustave Roussy, Villejuif, France.; 4 Faculty of Medicine, Paris Saclay University, Kremlin-Bicêtre, France.; 5 MOLIT Institute, Cancer Center Heilbronn-Franken, SLK-Clinics, Heilbronn, Germany.; 6 Department of Lung Cancer and Thoracic Tumors, Maria Sklodowska-Curie Memorial, National Research Institute of Oncology, Warsaw, Poland.; 7 Department of Oncology & Radiotherapy and Early Phase Clinical Trials Centre, Medical University of Gdańsk, Gdańsk, Poland.; 8 Phase 1 Unit, Fondazione Policlinico Universitario Agostino Gemelli IRCCS, Rome, Italy.; 9 Genentech, Inc., South San Francisco, California.; 10 Roche, Mississauga, Canada.; 11 Department of Internal Medicine, Henry Ford Cancer Institute/Henry Ford Health System, Detroit, Michigan.

## Abstract

**Purpose:**

Patients with tumor mutational burden (TMB)–high tumors can derive benefit from atezolizumab, though previous studies have used inconsistent TMB cutoffs. We report data for atezolizumab in patients with TMB-high solid tumors from the phase II TAPISTRY multicohort trial, using TMB cutoffs of ≥13 and ≥16 mutations per megabase (mut/Mb).

**Patients and Methods:**

Patients with PD-L1 inhibitor–naïve, TMB-high (≥13 mut/Mb), advanced/metastatic solid tumors received atezolizumab every 21 days [1,200 mg for adults, 15 mg/kg (up to 1,200 mg/kg) for children]. The primary endpoint was independent review committee (IRC)–assessed objective response rate (ORR) for TMB ≥16 mut/Mb. Secondary endpoints (using TMB ≥13 mut/Mb) included IRC-assessed ORR, duration of response (DOR), progression-free survival (PFS), and safety.

**Results:**

As of November 9, 2023 (median survival follow-up: 9.8 months), 148 patients received treatment. Median age was 63 years, 31.8% of patients had >2 prior therapy lines, and the most common tumor types were colorectal (29.1%), breast (8.8%), and gastroesophageal (8.8%). IRC-assessed ORR was 22.3% [95% confidence interval (CI), 15–31.2] with TMB ≥16 mut/Mb (*n* = 112), and 20.2% (95% CI, 13.6–28.1) with TMB ≥13 mut/Mb (*n* = 129). Median IRC-assessed DOR was not estimable. Median IRC-assessed PFS was 2.8 (95% CI, 1.7–5.4) and 2.7 (95% CI, 1.5–4.2) months using TMB ≥16 and ≥13 mut/Mb, respectively. Adverse events were reported in 93.2% of patients, of which 53.4% were treatment-related (no grade 5) and 40.5% were grade ≥3.

**Conclusions:**

Atezolizumab led to moderate antitumor activity in various TMB-high solid tumors. Safety was consistent with previous reports.

**Translational Relevance**

Checkpoint inhibitors (CPI) have transformed the treatment landscape for patients with different tumor types. However, not all patients respond to CPIs, resulting in an unmet need to identify those most likely to benefit. Previous reports describe some level of antitumor activity of atezolizumab in various tumor mutational burden (TMB)–high solid tumors. However, understanding the relevance of TMB as a predictive biomarker for response to CPIs is challenging, given the lack of standardized methodology and inconsistent use of TMB cutoffs. Cohort D of the phase II TAPISTRY trial was designed to investigate atezolizumab in TMB-high solid tumors using two TMB cutoffs: ≥13 and ≥16 mutations per megabase. We observed that antitumor activity was moderate with atezolizumab across a variety of TMB-high solid tumor types. Our findings support TMB-high status as a predictive biomarker for response to CPIs but warrant further research to identify populations with TMB-high tumors who would benefit most from atezolizumab.

## Introduction

Checkpoint inhibitors (CPI) have transformed the treatment landscape for patients with different tumor types (1, 2); however, not all patients respond to CPIs (3, 4). There remains an unmet need to identify patients who are most likely to benefit from CPI therapy, to enable more selective drug use and overcome treatment resistance (5).

Tumor mutational burden (TMB), defined as the number of somatic mutations per megabase (mut/Mb) of interrogated genomic sequence, is an emerging tumor-agnostic biomarker in several cancer types (5). Many CPI studies use different tumor/blood TMB cutoffs (6), and there is increasing evidence that patients with TMB-high tumors, defined as ≥10 mut/Mb in tumor samples, may benefit from CPI therapy (7), irrespective of the tumor type (8–10). In the KEYNOTE-158 phase II study, which enrolled patients with TMB-high, pretreated, unresectable/metastatic solid tumors, the anti–PD-1 antibody pembrolizumab was associated with a clinically meaningful objective response rate (ORR; ref. 11). Based on these data, pembrolizumab monotherapy was approved as a tumor-agnostic therapy by the U.S. Food and Drug Administration for the treatment of patients with TMB-high (≥10 mut/Mb) solid tumors, highlighting the significance of TMB as a biomarker for response to cancer immunotherapy (5, 12–15).

Studies have suggested that atezolizumab could be effective in patients with TMB-high solid tumors (14, 16, 17). In a retrospective analysis of data from seven atezolizumab monotherapy studies across multiple tumor types, elevated tumor TMB (≥16 mut/Mb) was associated with improved ORR and duration of response (DOR) compared with lower TMB status (<16 mut/Mb; ref. 16). In addition, an improved survival benefit was reported with atezolizumab in patients with non–small cell lung cancer (NSCLC) and elevated blood TMB (cutoffs of ≥10, ≥16, and ≥20) in a retrospective analysis of two randomized trials (17). A phase II study of atezolizumab as first-line therapy for NSCLC also reported a higher ORR in patients with elevated blood TMB (cutoff of ≥16; equivalent to approximately 14.5 mut/Mb) compared with lower blood TMB cutoffs (cutoff of <16; ref. 14). However, understanding the relevance of TMB as a predictive biomarker for response to atezolizumab and CPIs, in general, is challenging, given the lack of standardized methodology, including a consensus determining a cutoff for high TMB (6).

We report the efficacy and safety of atezolizumab in patients with TMB-high advanced or metastatic solid tumors from cohort D of the phase II tumor-agnostic TAPISTRY study (NCT04589845). We analyzed two TMB cutoffs: ≥16 mut/Mb, based on previous clinical trial experience with atezolizumab as outlined above, and ≥13 mut/Mb, according to the definition of TMB-high status by Foundation Medicine, Inc. (FMI) at the time of study inception (18).

## Patients and Methods

### Study design

TAPISTRY, a phase II, global, multicenter, open-label, multicohort study, evaluated the safety and efficacy of targeted therapies or immunotherapy as single agents or in specified combinations in patients with unresectable, locally advanced, or metastatic solid tumors harboring specific oncogenic genomic alterations or high TMB. Patients were assigned to treatment based on the presence of tumor genetic alterations, as identified by next-generation sequencing (NGS), that were known or expected to be predictive of response and/or clinical outcomes. To date, patients have been enrolled in 13 cohorts, each with specific endpoints (Supplementary Fig. S1).

The study protocol was approved by Institutional Review Boards at participating sites. The study was conducted in accordance with the Declaration of Helsinki and the International Council for Harmonisation guidelines for Good Clinical Practice. All patients provided written informed consent.

### Cohort D

Cohort D of the TAPISTRY study is a single-arm, open-label cohort designed to evaluate the safety and efficacy of atezolizumab in patients with TMB-high solid tumors (≥13 mut/Mb; Supplementary Fig. S2). To limit variability in TMB measurements, TMB-high tumors were determined by non-FMI local tissue TMB testing and FMI tissue testing on newly collected or archival tumor tissue (or blood when tissue was unavailable or insufficient) using protocol-specified FMI NGS assays [FoundationOne CDx, FoundationOne Heme, or FoundationOne Liquid CDx (FMI)]. Both pediatric and adult patients were eligible. Key inclusion criteria included measurable disease per Response Evaluation Criteria in Solid Tumors version 1.1 (RECIST v1.1); disease progression on prior treatment, or previously untreated disease with no available acceptable treatment; and Eastern Cooperative Oncology Group performance status 0 to 1 for adults, Karnofsky score ≥50% for children aged 16 to <18 years, or Lansky score ≥50% for children aged <16 years. Patients with symptomatic or actively progressing central nervous system (CNS) metastases or who had received prior PD-1/PD-L1 inhibitors were excluded. Complete eligibility criteria are provided in the Supplementary methods.

### Treatment

Patients received intravenous atezolizumab at 1,200 mg for adults (aged ≥18 years) and 15 mg/kg (maximum 1,200 mg) for children (aged <18 years) on day 1 of each 21-day cycle until disease progression, loss of clinical benefit as determined by the investigator, unacceptable toxicity, patient or physician decision to discontinue, or death.

### Endpoints

The primary study endpoint was independent review committee (IRC)–assessed ORR in patients with TMB ≥16 mut/Mb in the enrollment assay per RECIST v1.1. ORR was also assessed in the FMI blood and tissue assays and non-FMI local tissue assay subgroups. Secondary endpoints were assessed in patients with TMB ≥13 mut/Mb and TMB ≥16 mut/Mb and included IRC-assessed ORR (in patients with TMB ≥13 mut/Mb), DOR, clinical benefit rate (CBR), and progression-free survival (PFS); investigator-assessed ORR, DOR, CBR, and PFS; IRC and investigator-assessed time to CNS progression; overall survival (OS); and safety. An exploratory analysis of efficacy by PD-L1 expression status in the efficacy-evaluable population was also conducted.

### Assessments

Tissue and blood samples were collected at baseline to evaluate predictive and/or prognostic biomarkers including those related to driver oncogene signaling, response to study treatment, tumor pathogenesis, and mechanisms of resistance. Tumor assessments were performed at screening, every 6 weeks after day 1 of cycle 1 for the first year of treatment, and every 9 weeks thereafter. Tumor response was assessed using RECIST v1.1. All primary imaging data were centrally reviewed. Efficacy by PD-L1 status, determined by tumor proportion score (TPS) and combined positive score (CPS), and PD-L1 expression was assessed via immunohistochemistry (IHC) at a central laboratory using the PD-L1 IHC 22C3 pharmDx assay (RRID: AB_2889976; Agilent) in formalin-fixed tumor samples. Expression was categorized according to the TPS and CPS (19). Adverse events (AE), regardless of relationship to the study drug, were reported until 90 days after the final dose of study treatment or until the initiation of new systemic anticancer therapy; serious AEs (SAE) were reported beyond 90 days. The severity of AEs was graded according to the National Cancer Institute Common Terminology Criteria for Adverse Events version 5.0. An AE was classified as serious if it was fatal, was life-threatening, required or prolonged inpatient hospitalization, resulted in persistent or significant disability/incapacity, was a congenital anomaly/birth defect, or was deemed a significant medical event by the investigator.

### Statistical analysis

The safety-evaluable population comprised all patients who received ≥1 dose of atezolizumab. The efficacy-evaluable population included all safety-evaluable patients with TMB-high (≥13 mut/Mb per the FMI enrollment assay) primary extracranial solid tumors and measurable disease by RECIST v1.1 per IRC, who met cohort-specific eligibility criteria. ORR and 95% confidence intervals (CI) were calculated using the Clopper–Pearson method; the primary endpoint (IRC-assessed ORR in patients with TMB ≥16 mut/Mb) was compared with the prespecified threshold of 10% (the lower bound of the associated 95% CI excludes the standard-of-care ORR of 10%). ORR was defined as the proportion of patients with a complete response (CR) or partial response (PR) per RECIST v1.1. CBR was defined as the rate of patients with confirmed CR or PR or stable disease maintained for ≥24 weeks. The Kaplan–Meier method was used to estimate the median DOR, OS, and PFS with 95% CIs. Patients who experienced disease progression without CNS progression were censored for time to CNS progression at disease progression. All statistical analyses were performed using the statistical software SAS version 9.4.

## Results

### Patients

**Figure 1.**
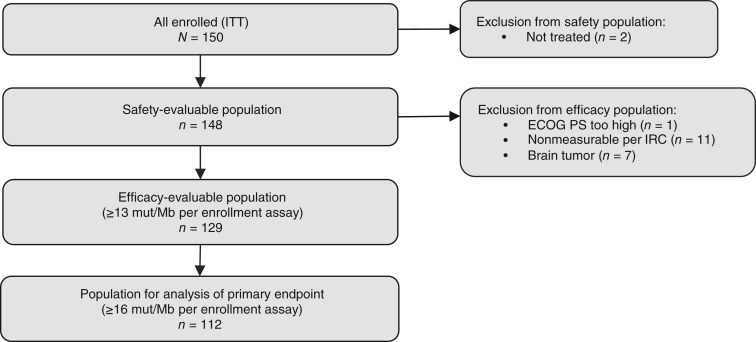
TAPISTRY cohort D flow diagram. Overall, 150 patients were enrolled (ITT). The safety-evaluable population consisted of 148 patients; 129 patients with ≥13 mut/Mb were included in the efficacy-evaluable population, and 112 patients with ≥16 mut/Mb were included in the analysis of the primary endpoint. ECOG PS, Eastern Cooperative Oncology Group performance status; ITT, intent to treat.

As of March 9, 2023, 150 patients with TMB ≥13 mut/Mb were enrolled in cohort D. At the data cutoff (November 9, 2023), 148 patients had received treatment and were included in the safety-evaluable population (Fig. 1). The efficacy-evaluable population included 129 patients with TMB-high (≥13 mut/Mb) tumors, with a total of 112 patients with TMB ≥16 mut/Mb included in the analysis of the primary endpoint of IRC-assessed ORR. The median age was 63 years (range: 11–86; one patient was aged <18 years), 56.1% of patients were male, and 68.2% had received 0 to 2 lines of prior therapy. The most common tumor types were colorectal (*n* = 43; 29.1%), breast (*n* = 13; 8.8%), and gastroesophageal (*n* = 13; 8.8%; Table 1). The representativeness of study participants is further contextualized in Supplementary Table S1.

**Table 1.** Baseline characteristics.

​	Atezolizumab (*N* = 148)
Median age, years (range)	63.0 (11–86)
<18/18–65/>65	1 (0.7)/90 (60.8)/57 (38.5)
Male	83 (56.1)
Race	​
White	92 (62.2)
Asian	37 (25)
Black/African American	2 (1.4)
American Indian/Alaska Native	1 (0.7)
Native Hawaiian or other Pacific Islander	1 (0.7)
Unknown	15 (10.1)
Baseline ECOG PS value	​
*n*	147
0/1/2	42 (28.6)/104 (70.7)/1 (0.7)
Number of prior lines of therapy	​
0–2/3–5/6–8/9+	101 (68.2)/33 (22.3)/10 (6.8)/4 (2.7)
Staging at study entry	​
*n*	147
IIB/IIIA/IIIB/IIIC/IV	1 (0.7)/1 (0.7)/1 (0.7)/1 (0.7)/143 (97.3)
Metastases present at study entry	​
CNS/liver/lung/bone	11 (7.4)/69 (46.6)/58 (39.2)/37 (25)
Cancer type	​
Colorectal cancer	43 (29.1)
Breast cancer	13 (8.8)
Gastroesophageal cancer	13 (8.8)
Hepatobiliary cancer	9 (6.1)
Prostate cancer	9 (6.1)
Occult primary or cancer of unknown primary	8 (5.4)
CNS/brain cancer	7 (4.7)
Non–small cell lung cancer	6 (4.1)
Endometrial/uterine cancer	5 (3.4)
Neuroendocrine and adrenal tumor	5 (3.4)
Nonmelanoma skin cancer	5 (3.4)
Cervical cancer	4 (2.7)
Head and neck cancer	4 (2.7)
Pancreatic cancer	4 (2.7)
Sarcoma	4 (2.7)
Small cell lung cancer	2 (1.4)
Bladder/urinary tract cancer	1 (0.7)
Kidney cancer	1 (0.7)
Melanoma	1 (0.7)
Ovarian cancer	1 (0.7)
Parathyroid carcinoma	1 (0.7)
Thymoma and thymic carcinoma	1 (0.7)
Thyroid cancer	1 (0.7)

Data are *n* (%) unless otherwise specified.

Abbreviation: CNS, central nervous system; ECOG PS, Eastern Cooperative Oncology Group performance score.

### Efficacy

After a median follow-up of 9.8 months, IRC-assessed ORR in patients with TMB ≥16 mut/Mb (primary endpoint) using the enrollment assay (*n* = 112) was 22.3% (95% CI, 15–31.2; Table 2). IRC-assessed ORR in patients with TMB status determined using the FMI tissue assay (*n* = 59) was 33.9% (95% CI, 22.1–47.4) versus 20.5% (95% CI, 12.2–31.2) for the non-FMI local tissue assay (*n* = 78) and 14.1% (95% CI, 6.6–25) for the FMI blood assay (*n* = 64). Median DOR was not estimable (NE) across all assays. Among the different assays, the CBR was 44.1% (95% CI, 31.2–57.6) for the FMI tissue assay versus 33% (95% CI, 24.4–42.6), 32.1% (95% CI, 21.9–43.6), and 18.8% (95% CI, 10.1–30.5) for the enrollment assay, non-FMI local tissue assay, and FMI blood assays, respectively. Median IRC-assessed PFS was 5.4 months (95% CI, 2.8–17.4) for the FMI tissue assay and ranged from 2.7 to 2.8 months with the enrollment assay, non-FMI local tissue assay, and FMI blood assay.

**Table 2.** IRC-assessed endpoints per RECIST v1.1 according to NGS assay type in patients with TMB ≥13 and ≥16 mut/Mb.

​	TMB ≥16 mut/Mb				TMB ≥13 mut/Mb			
	Enrollment assay^a^	FMI blood assay^b^	FMI tissue assay^c^	Non-FMI local tissue assay	Enrollment assay^a^	FMI blood assay^b^	FMI tissue assay^c^	Non-FMI local tissue assay
	(*n* = 112)	(*n* = 64)	(*n* = 59)	(*n* = 78)	(*n* = 129)	(*n* = 73)	(*n* = 77)	(*n* = 88)
ORR, % (95% CI)	22.3 (15–31.2)	14.1 (6.6–25)	33.9 (22.1–47.4)	20.5 (12.2–31.2)	20.2 (13.6–28.1)	15.1 (7.8–25.4)	27.3 (17.7–38.6)	19.3 (11.7–29.1)
Best objective response, *n* (%)	​	​	​	​	​	​	​	​
CR	4 (3.6)	1 (1.6)	3 (5.1)	2 (2.6)	4 (3.1)	1 (1.4)	3 (3.9)	2 (2.3)
PR	21 (18.8)	8 (12.5)	17 (28.8)	14 (17.9)	22 (17.1)	10 (13.7)	18 (23.4)	15 (17)
SD	38 (33.9)	23 (35.9)	20 (33.9)	26 (33.3)	43 (33.3)	23 (31.5)	25 (32.5)	29 (33)
PD	39 (34.8)	24 (37.5)	15 (25.4)	29 (37.2)	49 (38)	29 (39.7)	24 (31.2)	35 (39.8)
Missing	10 (8.9)	8 (12.5)	4 (6.8)	7 (9)	11 (8.5)	10 (13.7)	7 (9.1)	7 (8)
Median DOR, months (95% CI)	NE (20.8–NE)	NE (4.2–NE)	NE (20.8–NE)	NE (20.8–NE)	NE (20.8–NE)	NE (4.3–NE)	NE (20.8–NE)	NE (20.8–NE)
CBR^d^, % (95% CI)	33 (24.4–42.6)	18.8 (10.1–30.5)	44.1 (31.2–57.6)	32.1 (21.9–43.6)	30.2 (22.5–38.9)	19.2 (10.9–30.1)	37.7 (26.9–49.4)	30.7 (21.3–41.4)
Median PFS, months (95% CI)	2.8 (1.7–5.4)	2.7 (1.4–4.1)	5.4 (2.8–17.4)	2.8 (1.5–5.5)	2.7 (1.5–4.2)	1.7 (1.4–3)	3 (1.9–5.5)	2.7 (1.4–5.4)

Abbreviations: PD, progressive disease; SD, stable disease.

a FoundationOne CDx.

b FoundationOne Liquid CDx.

c FoundationOne Heme.

d Defined as patients with confirmed CR, PR, or SD for ≥24 weeks.

**Figure 2.**
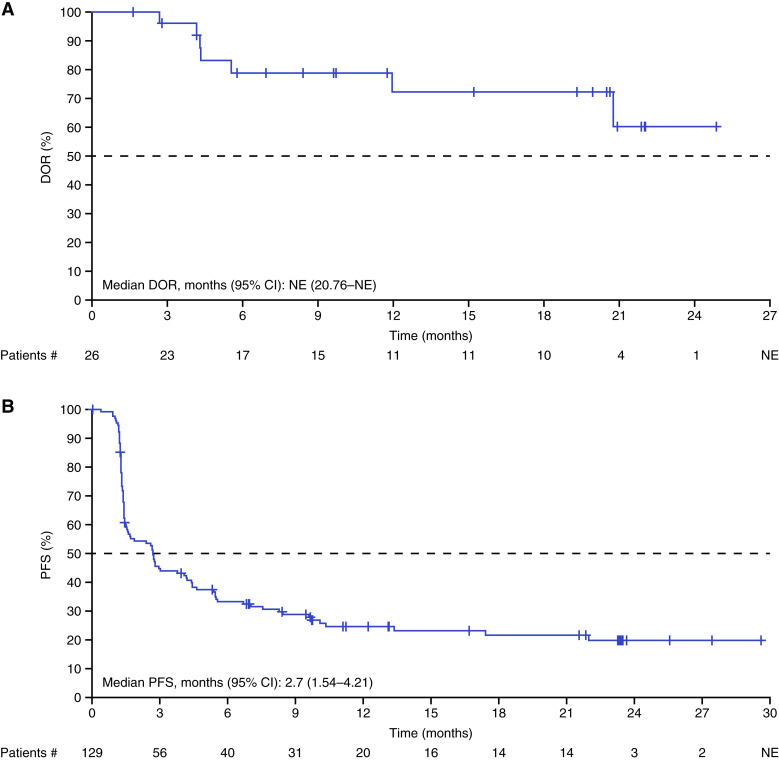
Kaplan–Meier plots of (**A**) confirmed DOR and (**B**) PFS, as assessed by IRC in patients with TMB ≥13 mut/Mb per the enrollment assay. When using a TMB cutoff of ≥13 mut/Mb per the enrollment assay (*n* = 129), median DOR was NE, and median IRC-assessed PFS was 2.7 months (95% CI, 1.5–4.2). #, Remaining patients at the corresponding time point; +, a censoring event.

When using a TMB cutoff of ≥13 mut/Mb per the enrollment assay (*n* = 129), the IRC-assessed ORR was 20.2% (95% CI, 13.6–28.1; Table 2), the median DOR was NE (Fig. 2A), and the CBR was 30.2% (95% CI, 22.5–38.9); the median IRC-assessed PFS was 2.7 months (95% CI, 1.5–4.2; Fig. 2B). Among patients with a TMB cutoff of ≥13 mut/Mb per the FMI tissue assay (*n* = 77), the IRC-assessed ORR was 27.3% (95% CI, 17.7–38.6), and the CBR was 37.7% (95% CI, 26.9–49.4). The median IRC-assessed PFS was 3 months (95% CI, 1.9–5.5) for the FMI tissue assay, 2.7 months for both the enrollment (95% CI, 1.5–4.2) and non-FMI local tissue (95% CI, 1.4–5.4) assays, and 1.7 months for the FMI blood assay (95% CI, 1.4–3).

Investigator-assessed efficacy endpoints for patients with TMB ≥13 and ≥16 mut/Mb are provided in Supplementary Table S2. An exploratory subgroup analysis of the most common tumor types (≥7 patients) found the following IRC-assessed ORR for patients with TMB ≥13 per the enrollment assay: occult primary tumors or cancer of unknown primary origin (*n* = 3/7), 42.9%; gastroesophageal (*n* = 4/11), 36.4%; prostate (*n* = 2/7), 28.6%; and colorectal (*n* = 5/40), 12.5%. An ORR of 0% was reported in patients with breast (*n* = 11) and hepatobiliary (*n* = 9) cancer (Table 3). Median time to IRC-assessed CNS progression, per RECIST v1.1, in patients with TMB ≥13 mut/Mb in the enrollment assay was 9.4 months (95% CI, 7.6–16.4). Median OS was 16.1 months (95% CI, 9.1–21.4) and 15 months (95% CI, 9.4–21.5) in patients with TMB ≥13 and ≥16 mut/Mb in the enrollment assay, respectively.

**Table 3.** Summary of IRC- and INV-assessed efficacy by most common (≥7 patients) tumor type in patients with TMB ≥13 mut/Mb per the enrollment assay.

​	Colorectal (*n* = 40)	Breast (*n* = 11)	Gastroesophageal (*n* = 11)	Hepatobiliary (*n* = 9)	Prostate (*n* = 7)	Occult primary or cancer of unknown primary (*n* = 7)
Median follow-up, months (range)	8.8 (1–31)	5.4 (1–23)	7.6 (4–24)	10.1 (2–26)	9.8 (2–24)	14.1 (1–25)
IRC-assessed	​	​	​	​	​	​
Best confirmed overall response, % (95% CI)	12.5 (4.2–26.8)	0 (0–28.5)	36.4 (10.9–69.2)	0 (0–33.6)	28.6 (3.7–71)	42.9 (9.9–81.6)
Median PFS, months (95% CI)	1.9 (1.4–4.1)	1.3 (1.2–2.7)	3 (1.5–NE)	4.6 (1.4–10.1)	2.8 (1.2–NE)	13.4 (1.4–NE)
Median DOR, months (95% CI)	NE (2.7–NE)	—	NE (4.3–NE)	—	—	12 (4.2–NE)
Median OS, months (95% CI)	11.3 (7.6–20.4)	6.4 (4.1–NE)	NE (4.8–NE)	10.1 (5.5–11.6)	16.4 (2.2–NE)	21.4 (12.4–NE)
INV-assessed	​	​	​	​	​	​
Best confirmed overall response, % (95% CI)	12.5 (4.2–26.8)	9.1 (0.2–41.3)	45.5 (16.8–76.6)	0 (0–33.6)	42.9 (9.9–81.6)	42.9 (9.9–81.6)
Median PFS, months (95% CI)	2.6 (1.4–4)	1.4 (1.3–2.8)	3.8 (2.7–NE)	5.5 (1.7–10.1)	5.5 (1.2–NE)	15.2 (3.9–NE)
Median DOR, months (95% CI)	NE (3–NE)	—	NE (2.8–NE)	—	—	13.8 (5.6–NE)
Median OS, months (95% CI)	11.3 (7.6–20.4)	6.4 (4.1–NE)	NE (4.8–NE)	10.1 (5.5–11.6)	16.4 (2.2–NE)	21.4 (12.4–NE)

Abbreviation: INV, investigator.

### Exploratory analyses of efficacy

Among patients with TMB ≥13 mut/Mb in the enrollment assay, ORR by IRC was 15.6% in patients with TPS <1% (*n* = 109) and 45.0% in patients with TPS ≥1% (*n* = 20); median DOR by IRC was NE and 20.8 months, respectively (Table 4). Median PFS by IRC was 2.6 months in patients with TPS <1% and 13.4 months in those with TPS ≥1%. By CPS analysis, ORR by IRC was 15.2% in patients with CPS <1% (*n* = 79) and 28.0% in patients with CPS ≥1% (*n* = 50); median DOR by IRC was NE in both populations. Median PFS by IRC was 2.7 months with both CPS cutoffs. Investigator-assessed efficacy endpoints per TPS and CPS <1% and ≥1% for patients with TMB ≥13 mut/Mb in the enrollment assay are presented in Supplementary Table S3.

**Table 4.** ORR, DOR, and PFS by IRC in patients with TMB ≥13 mut/Mb and TPS and CPS <1% and ≥1%.

​	TPS		CPS	
	<1% (*n* = 109)	≥1% (*n* = 20)	<1% (*n* = 79)	≥1% (*n* = 50)
ORR, % (95% CI)	15.6 (9.4–23.8)	45 (23.1–68.5)	15.2 (8.1–25)	28 (16.2–42.5)
Median DOR, months (95% CI)	NE (NE)	20.8 (12–NE)	NE (4.3–NE)	NE (12–NE)
Median PFS, months (95% CI)	2.6 (1.5–3)	13.4 (1.5–NE)	2.7 (1.5–4.2)	2.7 (1.5–5.5)

### Safety

In the safety-evaluable population (*n* = 148; median follow-up of 9.8 months), median treatment duration was 3.5 months (range, 0–31.4), and median dose intensity was 99.4% (range, 50.7–107.7). The majority of patients experienced an AE (*n* = 138; 93.2%), with 60 patients (40.5%) experiencing a grade ≥3 AE (Table 5). The most common AEs and grade ≥3 AEs can be found in Supplementary Tables S4 and S5. Four patients (2.7%) had grade 5 AEs (unexplained death, sepsis, renal failure, cardiac failure; *n* = 1 each), which were deemed not related to atezolizumab treatment. AEs led to study treatment discontinuation in six patients (4.1%) and to withdrawal from the study in three patients (2%).

**Table 5.** Safety overview.

Summary of AEs	Patients (*N* = 148)
Any AE	138 (93.2)
Grade 3–5 AE	60 (40.5)
AE with fatal outcome	4 (2.7)
Any AE leading to withdrawal from treatment	6 (4.1)
Any AE leading to withdrawal from the study	3 (2)
Any SAE	41 (27.7)
SAE leading to withdrawal from study	3 (2)
Any TRAE	79 (53.4)
Grade 3–5 TRAE	12 (8.1)
TRAE leading to withdrawal from study	0
TRAE with fatal outcome	0

Data are *n* (%) unless otherwise specified.

Treatment-related AEs (TRAE) occurred in 79 patients (53.4%; Table 5), most commonly (≥5% of patients) pruritus (*n* = 16; 10.8%), fatigue (*n* = 14; 9.5%), hypothyroidism (*n* = 14; 9.5%), increased alanine aminotransferase (ALT; *n* = 8; 5.4%), increased aspartate aminotransferase (AST; *n* = 8; 5.4%), and decreased appetite (*n* = 8; 5.4%). Grade ≥3 TRAEs were reported in 12 patients (8.1%) and comprised fatigue and abdominal pain (*n* = 2; 1.4% each) and one (0.7%) case each of diarrhea, vomiting, asthenia, pyrexia, adrenal insufficiency, hypophysitis, platelet count decrease, white blood cell count decrease, anemia, myocarditis, and cellulitis. No grade 5 TRAEs were reported, and no TRAEs led to withdrawal from the study.

SAEs were reported in 41 patients (28%), resulting in withdrawal from the study for three patients (2%; Table 5). The most common any-grade SAEs (≥2% of patients) were acute kidney injury, pyrexia, and pneumonia (*n* = 3; 2% each). Serious TRAEs occurred in nine patients (6.1%) and comprised pyrexia (*n* = 2; 1.4%) and abdominal pain, vomiting, fatigue, hemiparesis, nervous system disorder, myocarditis, hypophysitis, cellulitis, and infusion-related reaction (*n* = 1; 0.7% each).

AEs of special interest with atezolizumab occurred in 75 patients (50.7%), the most common (≥9% of patients) of which were hypothyroidism (*n* = 16; 10.8%) and increased ALT and increased AST (*n* = 14; 9.5% each; Supplementary Table S6).

## Discussion

In cohort D of the TAPISTRY study, atezolizumab monotherapy showed antitumor activity in patients with TMB-high solid tumors not previously treated with anti–PD-1/PD-L1 antibodies, with a confirmed ORR per IRC of 22.3% using a TMB cutoff of ≥16 mut/Mb. The ORR per IRC at a TMB cutoff of ≥13 mut/Mb was similar, at 20.2%. Median DOR per IRC was NE with either TMB cutoff.

Our results align with earlier studies investigating single-agent atezolizumab in patients with TMB-high tumors (14, 16, 17, 20–22). In a retrospective analysis using the FoundationOne assay for TMB of 987 patients with multiple tumor types, an ORR of 16.4% was reported with atezolizumab in the biomarker-evaluable population (BEP) versus 29.7% in patients with TMB-high tumors (≥16 mut/Mb); median DOR was 16.6 months in the BEP versus 29 months in the TMB-high subgroup (16). In addition, the phase IIa MyPathway multibasket study confirmed that the ORR with atezolizumab in patients with TMB-high (≥16 mut/Mb) advanced solid tumors was 38.1%, and the median DOR was not reached as of the data cutoff (21). Similarly, a retrospective analysis of the phase II IMvigor210 trial reported an ORR of 42% with atezolizumab in patients with advanced urothelial cancer and immunotherapy response score (IRS)–high tumors (a pan-tumor–based biomarker that incorporates TMB status) versus 10% in IRS-low tumors (22).

Although there is an association between higher TMB and clinical response for most cancers (14, 16), multiple factors can contribute to the variability of TMB estimates and reporting across assays (23). As a result, the mutational number defining TMB-high status can differ depending on the assay used, and a universally precise cutoff cannot be used to predict the likelihood of benefit from CPI treatment (24). FoundationOne CDx is the only FDA-approved panel assay for reporting TMB status, but others (MSK-IMPACT; NantHealth’s Omics Core test; PGDx elio) have been deemed substantially equivalent by the FDA (25). However, access to these panels is not universal, so additional NGS-targeted gene panel assays have been developed by local providers (25). The Friends of Cancer Research TMB Harmonization Project aimed to characterize the variability of 11 panel-based TMB estimates by directly comparing panel-based TMB estimates from participating laboratories (26). They identified that there was variability across panels, with some consistently over- and underestimating TMB (26). In TAPISTRY cohort D, we observed that clinical response may vary by the type of assay used to determine TMB status (6, 24), with numerically higher response rates seen when using the FMI tissue assay versus non-FMI local tissue and FMI blood assays. Among patients with TMB ≥13 mut/Mb per the enrollment assay, we also noted numerically higher response rates in patients with PD-L1 TPS or CPS ≥1% compared with PD-L1 TPS or CPS <1%. TPS testing is used to determine PD-L1 expression in NSCLC, with CPS utilized for other tumor types (27). Although patient numbers in the TMB ≥13 mut/Mb and TPS ≥1% subgroups were small (*n* = 20), our data suggest a possible role for TPS ≥1% as a marker of response to CPIs beyond NSCLC, and relatively low response rates in our study may be a result of a high proportion of patients with TPS <1%. Although PD-L1 expression is an independent biomarker for response to CPIs in NSCLC, TMB is known to complement the action of PD-L1; therefore, its inclusion in targeted NGS panels may enable a more accurate selection of patients most likely to benefit from CPI therapy (28, 29). However, due to the small sample sizes of the FMI blood and tissue assays, non-FMI local tissue assay, and PD-L1 TPS and CPS subgroups, interpretations of data should be made with caution.

In TAPISTRY cohort D, safety results were consistent with the known safety profile of atezolizumab monotherapy (30, 31), and no new safety signals were identified.

The strength of the TAPISTRY study is its multicohort platform design. It is extremely challenging to enroll a sufficiently large, randomized population of patients with rare genetic alterations for clinical trials investigating therapies that target rare genomic alterations found across a variety of tumors (32). This challenge is even more relevant for pediatric patients with solid tumors, which occur at a much lower incidence than adult tumors (33). The advantage of platform studies, such as TAPISTRY, is that they can overcome these barriers and enable the safety and efficacy of a targeted therapy to be evaluated in an age- and tumor-agnostic population.

We acknowledge there are some limitations in our study, mainly the variability in the calculation of TMB scores among the FMI and non-FMI local tissue assays, which may have affected the results. Only two TMB cutoffs were analyzed; therefore, our data cannot be generalized to all patients with TMB-high tumors. Additionally, the response to atezolizumab may have been affected by other factors in addition to TMB, such as comutations, concomitant medications, gut microbiota, human leukocyte antigen expression, and T-cell receptor repertoire (6). Specifically, microsatellite instability-high status, which has been shown to correlate with TMB-high status in patients with colorectal cancer (34), was not analyzed but could have contributed to tumor response. Although patients with active CNS metastases were excluded from the study, patients with stable CNS tumors were permitted, which could have affected the observed response rate, as CNS TMB-high tumors are typically not responsive to CPI therapy (35). Furthermore, the inclusion of a very broad range of tumor types resulted in only a small number of patients available for analysis with each tumor type, limiting the ability to compare therapeutic efficacy across tumor types. Although both adult and pediatric patients were eligible for study enrollment, only one pediatric patient received atezolizumab, limiting the assessment of treatment efficacy specifically in a pediatric population. Numerically higher response rates were also observed in the FMI tissue subgroup compared with the FMI blood subgroup; however, findings were limited by the small sample sizes. Additional studies could assess potential differences between tumor and blood TMB when treated with CPIs.

In summary, atezolizumab demonstrated moderate antitumor activity in patients with TMB-high solid tumors across a variety of tumor types. Safety findings were consistent with the known safety profile of atezolizumab. Further research may be warranted to identify populations with TMB-high tumors, including those with rare tumor types, who would benefit most from treatment with atezolizumab.

## Supplementary Material

Supplementary Methods S1 Supplementary methods - Eligibility Criteria

Supplementary Figure S1 Supplementary figure S1 - TAPISTRY overall study design

Supplementary Figure S2 Supplementary figure S2 - Study design for TAPISTRY Cohort D

Supplementary Table S1 Supplementary table S1 - representativeness of study participants

Supplementary Table S2 Supplementary table S2 - investigator-Assessed ORR, DOR, CBR, and PFS Per RECIST v1.1 in Patients with TMB ≥13 and ≥16 mut/Mb

Supplementary Table S3 Supplementary table S3 - ORR, DOR, and PFS by Investigator Assessment in Patients with TMB ≥13 mut/Mb and TPS and CPS <1% and ≥1%

Supplementary Table S4 Supplementary table S4 - Most Common (≥10% of Patients) Adverse Events

Supplementary Table S5 Supplementary table S5 - Most Common (≥2% of Patients) Grade ≥3 Adverse Events

Supplementary Table S6 Supplementary table S6 - Adverse Events of Special Interest

## Data Availability

For up-to-date details on Roche’s Global Policy on the Sharing of Clinical Information and how to request access to related clinical study documents, visit https://go.roche.com/data_sharing. Anonymized records for individual patients across more than one data source external to Roche cannot, and should not, be linked due to a potential increase in the risk of patient reidentification. For eligible studies, qualified researchers may request access to individual patient-level clinical data through a data request platform. At the time of writing, the request platform is Vivli (https://vivli.org/ourmember/roche/).
