# Supplementary material for: TAPISTRY: A Phase II Study of Atezolizumab in Patients with Tumor Mutational Burden–High Tumors
Source: Clin Cancer Res. 2026 Jan 9;32(6):1078–86. doi: 10.1158/1078-0432.CCR-25-3336 (PMC13012244; doi:10.1158/1078-0432.CCR-25-3336)
Supplement: Supplementary Methods S1 — Supplementary methods - Eligibility Criteria [file ccr-25-3336_supplementary_methods_s1_suppms1.docx]

# SUPPLEMENTARY METHODS

## Complete Eligibility Criteria for Cohort D

*Patients must meet the following general inclusion criteria to be eligible to enroll in any cohort:*

- For patients whose biomarker status is unknown and/or for patients with an ineligible local next-generation sequencing (NGS) test result: signed biomarker eligibility testing informed consent form and willingness to participate in an assigned cohort based on their identified oncogenic biomarker(s)
- For patients with a positive biomarker status: signed cohort-specific informed consent form
  - For pediatric patients: informed consent form must be signed by either the parent(s) or a legal representative
- For pediatric patients: signed child’s informed assent, when appropriate as determined by patient’s age and individual site and country standards
- Histologically or cytologically confirmed diagnosis of advanced and unresectable or metastatic solid malignancy
- Measurable disease as defined by Response Evaluation Criteria in Solid Tumors version 1.1, Response Assessment in Neuro-Oncology, or International Neuroblastoma Response Criteria
- Performance status as follows:
  - Patients aged ≥18 years: Eastern Cooperative Oncology Group (ECOG) performance status 0–1
  - Patients aged 16 to <18 years: Karnofsky score ≥50%
  - Patients aged <16 years: Lansky score ≥50%
- For patients aged ≥18 years: adequate hematologic and end-organ function as defined below:
  - Absolute neutrophil count (ANC) ≥1000/μL
  - Platelet count ≥100 x10^9^/L
  - Hemoglobin ≥8 g/dL
  - Total bilirubin ≤1.5 x upper limit of normal (ULN), with the following exception: patients with known Gilbert’s syndrome who have a serum bilirubin ≤3 x ULN may be enrolled
  - Aspartate aminotransferase (AST) and alanine aminotransferase (ALT) ≤3
    x ULN, with the following exception: patients with liver metastases who have an AST or ALT ≤5 x ULN may be enrolled
- For patients aged <18 years: adequate hematologic and end-organ function as defined below:
  - ANC ≥1000/μL (without transfusion)
  - Platelet count ≥75 x 10^9^/L (without transfusion)
  - Hemoglobin ≥8 g/dL (transfusion permitted)
  - Bilirubin ≤1.5 x ULN for age
    - Patients with known Gilbert’s syndrome who have a serum bilirubin ≤3 x ULN may be enrolled
  - AST and ALT ≤3 x ULN for age
  - Serum creatinine ≤1.5 x ULN for age or creatinine clearance (or radioisotope glomerular filtration rate) >70 mL/min/1.73 m^2^
  - International normalized ratio (INR) and activated partial thromboplastin time (aPTT) ≤1.5 x ULN for age
- Disease progression on prior treatment, or previously untreated disease with no available acceptable treatment
- Adequate recovery from most recent systemic or local treatment for cancer
- Life expectancy ≥8 weeks
- Ability to comply with the study protocol, in the investigator's judgment
- For female patients of childbearing potential: negative serum pregnancy test ≤7 days prior to initiating study treatment, agreement to remain abstinent or use single or combined contraception methods that result in a failure rate of <1% per year for the period defined in the cohort-specific inclusion criteria, and agreement to refrain from donating eggs during the same period
  - Examples of contraception methods with a failure rate of <1% per year: vasectomy, bilateral tubal ligation, intrauterine device, or intrauterine hormone-releasing system
    - Barrier methods such as condoms, diaphragms, caps (with spermicide), or sponges (with spermicide) have a failure rate of <1% per year. Contraception methods that have a failure rate of >1% per year must be combined with a contraception method that has a failure rate of <1% per year
    - The use of oral contraceptives should be supplemented with a barrier method (preferably a male condom)
  - A female is considered to be of childbearing potential if she is postmenarcheal, has not reached a postmenopausal state (≥12 continuous months of amenorrhea with no identified cause other than menopause), and has not undergone surgical sterilization (removal of ovaries and/or uterus). The definition of childbearing potential may be adapted for alignment with local guidelines or requirements
  - The reliability of sexual abstinence should be evaluated in relation to the duration of the clinical trial and the preferred and usual lifestyle of the patient. Periodic abstinence (e.g., calendar, ovulation, symptothermal, or postovulation methods) and withdrawal are not acceptable
- For male patients: Willingness to remain abstinent or use acceptable methods of contraception as defined in the cohort-specific inclusion criteria

*Patients must meet the following additional inclusion criteria for entry into Cohort D:*

- Tumor mutational burden (TMB)-high status, as determined by Foundation Medicine, Inc. NGS assays (FoundationOne CDx, FoundationOne Heme, or FoundationOne Liquid CDx, which may be considered investigational per local regulations) either through local testing or Sponsor-supported eligibility testing. Non-Foundation Medicine local NGS assays (tissue or blood) are not allowed for determining biomarker eligibility
  - TMB-high is defined as ≥13 mutations per megabase
- For females of childbearing potential: negative pregnancy test ≤7 days prior to initiating study treatment; agreement to remain abstinent (refrain from heterosexual intercourse) or use single or combined contraception methods that result in a failure rate of <1% per year during the treatment period and for ≥5 months after the last dose of atezolizumab; and agreement to refrain from donating eggs during this same period
- For patients with primary central nervous system (CNS) tumors: current treatment with a stable dose of anticonvulsants and corticosteroids
- Negative HIV test at screening
  - Sites should include an HIV test during screening, as allowed per local regulations
- Negative hepatitis B surface antigen test at screening
- Positive hepatitis B surface antibody (HBsAb) test at screening, or negative HBsAb at screening accompanied by either of the following:
  - Negative total hepatitis B core antibody (HBcAb)
  - Positive total HBcAb test followed by a negative (per local laboratory definition) hepatitis B virus (HBV) DNA test
- Negative hepatitis C virus (HCV) antibody test at screening, or positive HCV antibody test followed by negative HCV RNA test at screening
  - The HCV RNA test will be performed only for patients who have a positive HCV antibody test
- For patients aged ≥18 years: adequate hematologic and end-organ function, defined by the following laboratory test results, obtained within 14 days prior to initiation of study treatment:
  - ANC ≥1500/µL without granulocyte colony-stimulating factor support
  - Lymphocyte count ≥500/µL
  - Platelet count ≥100×10^9^/L without transfusion
  - AST, ALT, and alkaline phosphatase (ALP) ≤2.5 x ULN, with the following exceptions:
    - Patients with documented liver metastases: AST and ALT ≤5 x ULN
    - Patients with documented liver or bone metastases: ALP ≤5 x ULN
  - Serum albumin ≥25 g/L (2.5 g/dL)
  - For patients not receiving therapeutic anticoagulation: INR and aPTT ≤1.5 x ULN
  - For patients receiving therapeutic anticoagulation: stable anticoagulant regimen
  - Serum creatinine ≤1.5 x ULN or calculated creatinine clearance ≥40 mL/min (using the Cockcroft-Gault equation)

*Patients who meet any of the following criteria will be ineligible for NGS biomarker eligibility testing (if required) and excluded from study entry in any cohort:*

- Current participation or enrollment in another therapeutic clinical trial
- Any anti-cancer treatment within 2 weeks prior to start of study treatment
- Whole brain radiotherapy within 14 days prior to start of study treatment
- Stereotactic radiosurgery within 7 days prior to start of study treatment
- Pregnant or breastfeeding, or intending to become pregnant during the study
- History of or concurrent serious medical condition or abnormality in clinical laboratory tests that, in the investigator's judgment, precludes the patient's safe participation in and completion of the study or confounds the ability to interpret data from the study
- Incomplete recovery from any surgery prior to the start of study treatment that would interfere with the determination of safety or efficacy of study treatment
- Significant cardiovascular disease, such as New York Heart Association cardiac disease (class II or higher), myocardial infarction, or cerebrovascular accident within 3 months prior to enrollment, unstable arrhythmias, or unstable angina
- History of another active cancer within 5 years prior to screening that may interfere with the determination of safety or efficacy of study treatment with respect to the qualifying solid tumor malignancy

*Patients who meet any of the following criteria will be excluded from entry into Cohort D:*

- Symptomatic or actively progressing CNS metastases
  - Patients with primary CNS tumors may enroll with actively progressing CNS metastases if they demonstrate stable corticosteroid dose within 4 weeks of study treatment initiation and no history of intracranial hemorrhage or spinal cord hemorrhage
  - Asymptomatic patients with treated or untreated CNS metastases are eligible, provided that all of the following criteria are met:
    - No ongoing requirement for corticosteroids as therapy for CNS metastases
    - No evidence of interim progression between completion of CNS-directed therapy and screening radiographic study
    - No history of intracranial hemorrhage or spinal cord hemorrhage
- History of leptomeningeal disease
- Primary CNS tumors with any of the following characteristics:
  - History of intracranial hemorrhage or spinal cord hemorrhage
  - Neurosurgical resection or brain biopsy of the primary brain tumor within 28 days of cycle 1, day 1
- Uncontrolled tumor-related pain
  - Patients requiring pain medication must be on a stable regimen at study entry
  - Symptomatic lesions (e.g., bone metastases or metastases causing nerve impingement) amenable to palliative radiotherapy should be treated prior to enrollment. Patients should be recovered from the effects of radiation. There is no required minimum recovery period
  - Asymptomatic metastatic lesions that would likely cause functional deficits or intractable pain with further growth (e.g., epidural metastasis that is not currently associated with spinal cord compression) should be considered for loco-regional therapy if appropriate prior to enrollment
- Uncontrolled pleural effusion, pericardial effusion, or ascites requiring recurrent drainage procedures twice monthly or more frequently
  - Indwelling pleural or abdominal catheters may be allowed provided the patient has adequately recovered from the procedure, is hemodynamically stable and symptomatically improved. The Medical Monitor is available to advise as needed
  - Patients with indwelling catheters (e.g., PleurX^®^) are allowed
- Uncontrolled or symptomatic hypercalcemia (ionized calcium >1.5 mmol/L, calcium >12 mg/dL, or corrected calcium >ULN)
- Active or history of autoimmune disease or immune deficiency, including, but not limited to, myasthenia gravis, myositis, autoimmune hepatitis, systemic lupus erythematosus, rheumatoid arthritis, inflammatory bowel disease, antiphospholipid antibody syndrome, Wegener granulomatosis, Sjögren syndrome, Guillain-Barré syndrome, or multiple sclerosis, with the following exceptions:
  - Patients with a history of autoimmune-related hypothyroidism who are on thyroid-replacement hormone are eligible for the study
  - Patients with controlled Type 1 diabetes mellitus who are on an insulin regimen are eligible for the study
  - Patients with eczema, psoriasis, lichen simplex chronicus, or vitiligo with

dermatologic manifestations only (e.g., patients with psoriatic arthritis are

excluded) are eligible for the study provided all of following conditions are met:

- - - Rash must cover <10% of body surface area
    - Disease is well-controlled at baseline and requires only low-potency topical corticosteroids
    - No occurrence of acute exacerbations of the underlying condition requiring psoralen plus ultraviolet A radiation, methotrexate, retinoids, biologic agents, oral calcineurin inhibitors, or high-potency or oral corticosteroids within the previous 12 months
- History of idiopathic pulmonary fibrosis, organizing pneumonia (e.g., bronchiolitis obliterans), drug-induced pneumonitis, or idiopathic pneumonitis, or evidence of active pneumonitis on screening chest CT scan
  - History of radiation pneumonitis in the radiation field (fibrosis) is permitted
- Active tuberculosis
- Severe infection within 4 weeks prior to initiation of study treatment, including, but not limited to, hospitalization for complications of infection, bacteremia, or severe pneumonia or any active infection that, in the opinion of the investigator, could impact patient safety
- Investigational therapy and prior anti-cancer therapy is prohibited within 4 weeks prior to initiation of study treatment and during study treatment
- Treatment with therapeutic oral or IV antibiotics within 2 weeks prior to initiation of study treatment
  - Patients receiving prophylactic antibiotics (e.g., to prevent a urinary tract infection or chronic obstructive pulmonary disease [COPD] exacerbation) are eligible for the study
- Prior allogeneic stem cell or solid organ transplantation
- Treatment with a live, attenuated vaccine within 4 weeks prior to initiation of study treatment, or anticipation of need for such a vaccine during atezolizumab treatment or within 5 months after the final dose of atezolizumab
- Current treatment with anti-viral therapy for HBV
- Prior treatment with CD137 agonists or immune checkpoint blockade therapies, including anti-programmed cell death protein 1 (PD-1), and anti-programmed death-ligand 1 (PD-L1) therapeutic antibodies
  - Patients who have had prior anti-cytotoxic T-lymphocyte-associated protein 4 (CTLA-4) treatment may be enrolled, provided the following requirements are met:
    - Last dose of anti-CTLA-4 ≥6 weeks prior to enrolment
    - No history of severe immune-related adverse events from the anti-CTLA-4 (National Cancer Institute Common Terminology Criteria for Adverse Events Grade 3 and 4)
- Treatment with systemic immunostimulatory agents including, but not limited to, interferons (IFN) and interleukin-2 (IL-2) within 4 weeks or 5 drug-elimination half-lives (whichever is longer) prior to initiation of study treatment
- Treatment with systemic immunosuppressive medication (including, but not limited to, corticosteroids, cyclophosphamide, azathioprine, methotrexate, thalidomide, and anti-tumor necrosis factor [TNF]-α agents) within 2 weeks prior to initiation of study treatment, or anticipation of need for systemic immunosuppressive medication during study treatment, with the following exceptions:
  - Patients who received acute, low-dose systemic immunosuppressant medication or a one-time pulse dose of systemic immunosuppressant medication (e.g., 48 hours of corticosteroids for a contrast allergy) are eligible for the study
  - Patients who received mineralocorticoids (e.g., fludrocortisone), corticosteroids for COPD or asthma, or low-dose corticosteroids for orthostatic hypotension or adrenal insufficiency are eligible for the study
- History of severe allergic anaphylactic reactions to chimeric or humanized antibodies or fusion proteins
- History of hypersensitivity to Chinese hamster ovary cell products
- History of hypersensitivity to atezolizumab or any of its excipients
