# Supplementary material for: TAPISTRY: A Phase II Study of Atezolizumab in Patients with Tumor Mutational Burden–High Tumors
Source: Clin Cancer Res. 2026 Jan 9;32(6):1078–86. doi: 10.1158/1078-0432.CCR-25-3336 (PMC13012244; doi:10.1158/1078-0432.CCR-25-3336)
Supplement: Supplementary Figure S1 — TAPISTRY overall study design [file ccr-25-3336_supplementary_figure_s1_suppfs1.docx]

**Supplementary Figure S1:** TAPISTRY overall study design


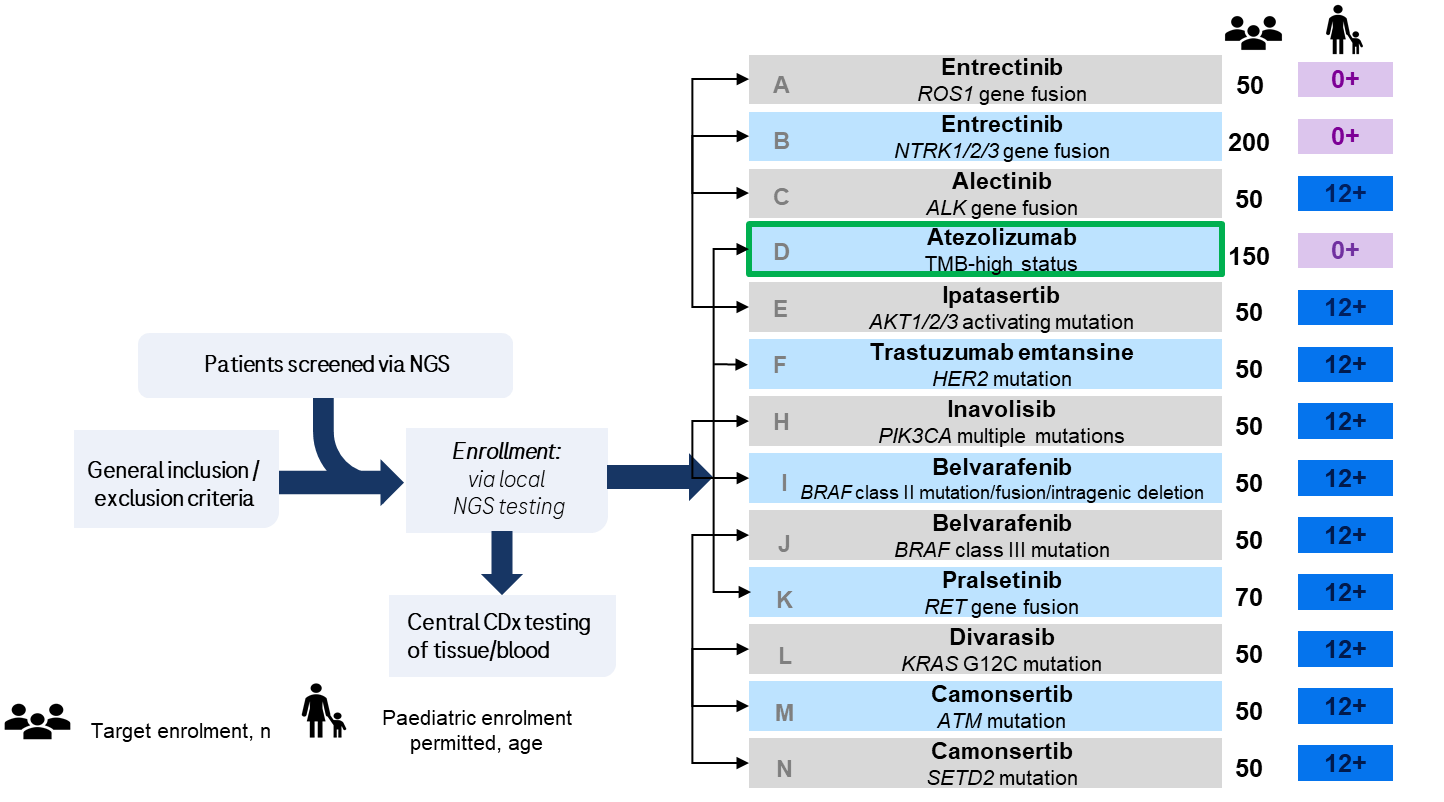


*ALK,* anaplastic lymphoma kinase; *AKT1/2/3,* Ak strain transforming gene 1/2/3; *ATM,* ataxia telangiectasia mutated; *BRAF,* V-Raf Murine Sarcoma Viral Oncogene Homolog B; CDx, companion diagnostic; *HER2,* human epidermal growth factor receptor 2; *KRAS G12C,* Kristen Rat Sarcoma Viral oncogene homolog G12C; *PIK3CA,* phosphatidylinositol-4,5-bisphosphate 3-kinase catalytic subunit alpha; NGS, next-generation sequencing; *NTRK1/2/3,* neurotrophic receptor tyrosine kinase genes 1/2/3; *RET,* rearranged during transfection; *ROS1,* ROS proto-oncogene 1; *SETD2*, SET domain containing 2 histone lysine methyltransferase; TMB, tumor mutational burden.
