# Supplementary material for: TAPISTRY: A Phase II Study of Atezolizumab in Patients with Tumor Mutational Burden–High Tumors
Source: Clin Cancer Res. 2026 Jan 9;32(6):1078–86. doi: 10.1158/1078-0432.CCR-25-3336 (PMC13012244; doi:10.1158/1078-0432.CCR-25-3336)
Supplement: Supplementary Figure S2 — Study design for TAPISTRY Cohort D [file ccr-25-3336_supplementary_figure_s2_suppfs2.docx]

**Supplementary Figure S2:** Study design for TAPISTRY Cohort D.


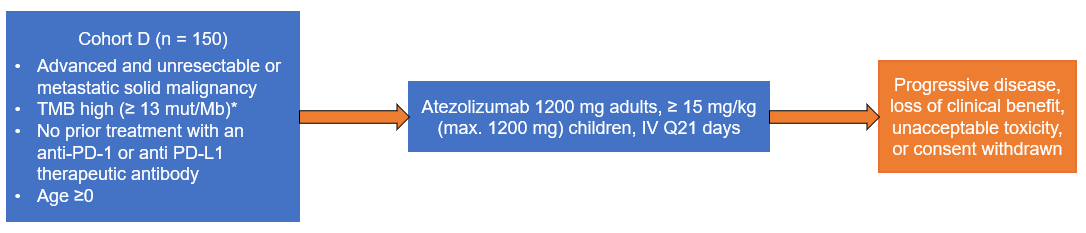


IV, intravenous, mut/Mb, mutations per megabase; PD-1, programmed death 1; PD-L1; programmed death-ligand 1; Q21 days, every 21 days; TMB, tumor mutational burden.
