# Supplementary material for: TAPISTRY: A Phase II Study of Atezolizumab in Patients with Tumor Mutational Burden–High Tumors
Source: Clin Cancer Res. 2026 Jan 9;32(6):1078–86. doi: 10.1158/1078-0432.CCR-25-3336 (PMC13012244; doi:10.1158/1078-0432.CCR-25-3336)
Supplement: Supplementary Table S1 — representativeness of study participants [file ccr-25-3336_supplementary_table_s1_suppts1.docx]

**Supplementary Table S1:** Representativeness of Study Participants

| **Cancer type(s)/subtype(s)/stage(s)/condition** | Solid tumors |
| --- | --- |
| **Considerations related to:** | |
| **Sex** | Globally, cancer prevalence is approximately 51% in males and 49% in females. Lung cancer is the most prevalent solid tumor across both sexes; however, breast cancer remains the most common cancer among women, while lung and prostate cancer predominate among men (1). |
| **Age** | Age is a significant factor influencing the development of solid tumors. Cancer incidence increases with age, likely due to the accumulation of age-related risk factors and reduced efficiency of cellular repair mechanisms (2). The median age at cancer diagnosis is 67 years, though this varies by cancer type — 63 years for breast cancer, 66 years for colorectal cancer, 71 years for lung cancer, and 68 years for prostate cancer (3). |
| **Race/ethnicity** | The incidence of solid tumors varies across racial and ethnic groups. The estimated lifetime risk of melanoma is 3% among White individuals, 0.1% among Black individuals, and 0.5% among Hispanic individuals (4). In the UK, melanoma, esophageal, bladder, and lung cancers are more common among White individuals compared with those from Black, Asian, or Mixed ethnic backgrounds (5). In contrast, prostate, myeloma, stomach, liver and womb cancers occur more frequently in Black individuals, and liver cancers are also more common in Asian populations (5). |
| **Geography** | Cancer is the leading cause of death worldwide (2). Certain solid tumors exhibit distinct geographical patterns; for example, colorectal cancer is far more common in Western countries (e.g., Australia/New Zealand) than in regions such as western Africa (6). This disparity is largely attributed to lifestyle and dietary factors, as shown by the rapid rise in incidence among migrants from low-risk to high-risk regions (6). |
| **Other considerations** | Genetic predispositions, environmental and occupational exposures (e.g., pollutants), access to healthcare, cultural influences, and socioeconomic factors all contribute to disparities in the development and outcomes of solid tumors (7). |
| **Overall study representativeness** | Cohort D of this phase II study included 150 patients. There were slightly more male than female participants, and the cohort represented a wide range of primary tumor types, including colorectal, breast, and gastroesophageal cancers. Eligible patients who met the study criteria and provided written informed consent were enrolled. The median age of participants was 63 years |

1. Worldwide Cancer Data. Word Cancer Research Fund. Accessed October 14, 2025. <https://www.wcrf.org/preventing-cancer/cancer-statistics/worldwide-cancer-data/#global-cancer-incidence-both-sexes>
2. Cancer. Word Health Organization. Accessed October 14, 2025. <https://www.who.int/news-room/fact-sheets/detail/cancer>
3. Age and Cancer Risk. National Cancer Institute. Accessed October 14, 2025. <https://www.cancer.gov/about-cancer/causes-prevention/risk/age>
4. Key Statistics for Melanoma Skin Cancer. American Can Society. Accessed October 14, 2025. <https://www.cancer.org/cancer/types/melanoma-skin-cancer/about/key-statistics.html>
5. First data in a decade highlights ethnic disparities in cancer. Cancer Research UK. Accessed October 14, 2025. <https://news.cancerresearchuk.org/2022/03/02/first-data-in-a-decade-highlights-ethnic-disparities-in-cancer/>
6. Geography and cancer. EBSCO. Accessed October 14, 2025. <https://www.ebsco.com/research-starters/health-and-medicine/geography-and-cancer>
7. Cancer Disparities. National Cancer Institute. Accessed October 14, 2025. <https://www.cancer.gov/about-cancer/understanding/disparities>
