# Supplementary material for: TAPISTRY: A Phase II Study of Atezolizumab in Patients with Tumor Mutational Burden–High Tumors
Source: Clin Cancer Res. 2026 Jan 9;32(6):1078–86. doi: 10.1158/1078-0432.CCR-25-3336 (PMC13012244; doi:10.1158/1078-0432.CCR-25-3336)
Supplement: Supplementary Table S2 — investigator-Assessed ORR, DOR, CBR, and PFS Per RECIST v1.1 in Patients with TMB ≥13 and ≥16 mut/Mb [file ccr-25-3336_supplementary_table_s2_suppts2.docx]

**Supplementary Table S2:** Investigator-Assessed ORR, DOR, CBR, and PFS Per RECIST v1.1 in Patients with TMB ≥13 and ≥16 mut/Mb

|  | **TMB ≥13 mut/Mb (n = 129)** | **TMB ≥16 mut/Mb (n = 112)** |
| --- | --- | --- |
| ORR, %  (95% CI) | 23.3  (16.3–31.5) | 25.9  (18.1–35.0) |
| Median DOR, months (95% CI), months | NE  (NE) | NE  (NE) |
| CBR^a^, %  (95% CI) | 32.6  (24.6–41.4) | 34.8  (26.1–44.4) |
| Median PFS, months (95% CI), | 2.8  (1.9–4.4) | 3.8  (2.6–5.5) |

^a^Defined as patients with confirmed CR, PR, or stable disease for ≥24 weeks.

CBR, clinical benefit rate; CI, confidence interval; CR, complete response; DOR, duration of response; FMI, Foundation Medicine, Inc; IRC, independent review committee; mut/Mb, mutations/megabase; NE, not estimable; ORR, objective response rate; PFS, progression-free survival; PR, partial response; RECIST, Response Evaluation Criteria in Solid Tumors; TMB, tumor mutational burden.
