# Supplementary material for: TAPISTRY: A Phase II Study of Atezolizumab in Patients with Tumor Mutational Burden–High Tumors
Source: Clin Cancer Res. 2026 Jan 9;32(6):1078–86. doi: 10.1158/1078-0432.CCR-25-3336 (PMC13012244; doi:10.1158/1078-0432.CCR-25-3336)
Supplement: Supplementary Table S3 — ORR, DOR, and PFS by Investigator Assessment in Patients with TMB ≥13 mut/Mb and TPS and CPS <1% and ≥1% [file ccr-25-3336_supplementary_table_s3_suppts3.docx]

**Supplementary Table S3:** ORR, DOR, and PFS by Investigator Assessment in Patients with TMB ≥13 mut/Mb and TPS and CPS <1% and ≥1%

|  | **TPS** | | **CPS** | |
| --- | --- | --- | --- | --- |
|  | **<1% (n = 109)** | **≥1% (n = 20)** | **<1% (n = 79)** | **≥1% (n = 50)** |
| ORR, %  (95% CI) | 21.1 (13.9–30.0) | 35.0 (15.4–59.2) | 22.8 (14.1–33.6) | 24.0 (13.1–38.2) |
| Median DOR, months (95% CI) | NE  (NE) | NE  (13.8–NE) | NE  (NE) | NE  (13.8–NE) |
| Median PFS, months (95% CI) | 2.7 (1.5–4.2) | 15.2  (2.8–NE) | 2.8  (1.5–5.5) | 3.9  (1.9–5.2) |

CI, confidence interval; CPS, combined positive score; DOR, duration of response; mut/Mb, mutations/megabase; NE, not estimable; ORR, objective response rate; PFS, progression-free survival; TMB, tumor mutational burden; TPS, tumor proportion score.
