# Supplementary material for: TAPISTRY: A Phase II Study of Atezolizumab in Patients with Tumor Mutational Burden–High Tumors
Source: Clin Cancer Res. 2026 Jan 9;32(6):1078–86. doi: 10.1158/1078-0432.CCR-25-3336 (PMC13012244; doi:10.1158/1078-0432.CCR-25-3336)
Supplement: Supplementary Table S4 — Most Common (≥10% of Patients) Adverse Events [file ccr-25-3336_supplementary_table_s4_suppts4.docx]

**Supplementary Table S4:** Most Common (≥10% of Patients) Adverse Events

|  | **Atezolizumab**  **(N=148)** |
| --- | --- |
| Fatigue | 33 (22.3) |
| Anemia | 29 (19.6) |
| Decreased appetite | 23 (15.5) |
| Pyrexia | 23 (15.5) |
| Arthralgia | 21 (14.2) |
| Diarrhea | 21 (14.2) |
| Pruritus | 20 (13.5) |
| Nausea | 18 (12.2) |
| Abdominal pain | 17 (11.5) |
| Constipation | 17 (11.5) |
| COVID-19 | 16 (10.8) |
| Hypothyroidism | 16 (10.8) |
| Vomiting | 15 (10.1) |

Data are n (%) unless otherwise specified.
