# Supplementary material for: TAPISTRY: A Phase II Study of Atezolizumab in Patients with Tumor Mutational Burden–High Tumors
Source: Clin Cancer Res. 2026 Jan 9;32(6):1078–86. doi: 10.1158/1078-0432.CCR-25-3336 (PMC13012244; doi:10.1158/1078-0432.CCR-25-3336)
Supplement: Supplementary Table S5 — Most Common (≥2% of Patients) Grade ≥3 Adverse Events [file ccr-25-3336_supplementary_table_s5_suppts5.docx]

**Supplementary Table S5:** Most Common (≥2% of Patients) Grade ≥3 Adverse Events

|  | **Atezolizumab**  **(N=148)** |
| --- | --- |
| Anemia | 10 (6.8) |
| Fatigue | 5 (3.4) |
| Abdominal pain | 4 (2.7) |
| ALT increased | 4 (2.7) |
| Pneumonia | 4 (2.7) |
| Acute kidney injury | 3 (2.0) |
| Ascites | 3 (2.0) |
| Diarrhea | 3 (2.0) |

Data are n (%) unless otherwise specified. Four patients (2.7%) had grade 5 adverse events (n=1 each of unexplained death, sepsis, renal failure, and cardiac failure).
ALT, alanine aminotransferase.
