# Supplementary material for: TAPISTRY: A Phase II Study of Atezolizumab in Patients with Tumor Mutational Burden–High Tumors
Source: Clin Cancer Res. 2026 Jan 9;32(6):1078–86. doi: 10.1158/1078-0432.CCR-25-3336 (PMC13012244; doi:10.1158/1078-0432.CCR-25-3336)
Supplement: Supplementary Table S6 — Adverse Events of Special Interest [file ccr-25-3336_supplementary_table_s6_suppts6.docx]

**Supplementary Table S6:** Adverse Events of Special Interest

|  | **Atezolizumab**  **(N=148)** |
| --- | --- |
| **Total number of patients with at least one AESI** | **75 (50.7)** |
| **Immune-mediated hepatitis  (diagnosis and lab abnormalities)** | **30 (20.3)** |
| ALT increased | 14 (9.5) |
| AST increased | 14 (9.5) |
| Ascites | 6 (4.1) |
| Blood bilirubin increased | 6 (4.1) |
| Hyperbilirubinemia | 5 (3.4) |
| GGT increased | 4 (2.7) |
| Hepatic cytolysis | 3 (2.0) |
| Bilirubin conjugated increased | 2 (1.4) |
| Blood bilirubin unconjugated increased | 2 (1.4) |
| Hypertransaminasemia | 2 (1.4) |
| Gastric varices | 1 (0.7) |
| Transaminases increased | 1 (0.7) |
| Varices esophageal | 1 (0.7) |
| **Immune-mediated hypothyroidism** | **18 (12.2)** |
| Hypothyroidism | 16 (10.8) |
| Autoimmune thyroiditis | 1 (0.7) |
| Immune-mediated thyroiditis | 1 (0.7) |
| **Immune-mediated rash** | **17 (11.5)** |
| Rash | 6 (4.1) |
| Rash maculo-papular | 5 (3.4) |
| Skin ulcer | 3 (2.0) |
| Erythema | 1 (0.7) |
| Rash erythematous | 1 (0.7) |
| Rash macular | 1 (0.7) |
| Rash papular | 1 (0.7) |
| Seborrheic dermatitis | 1 (0.7) |
| **COVID-19** | **16 (10.8)** |
| COVID-19 | 16 (10.8) |
| **Infusion related reactions** | **7 (4.7)** |
| Infusion related reaction | 7 (4.7) |
| **Immune-mediated hyperthyroidism** | **3 (2.0)** |
| Hyperthyroidism | 3 (2.0) |
| **Immune-mediated myositis** | **2 (1.4)** |
| Myopathy | 1 (0.7) |
| Myositis | 1 (0.7) |
| **Immune-mediated pericardial disorders** | **2 (1.4)** |
| Pericardial effusion | 1 (0.7) |
| Pericarditis | 1 (0.7) |
| **Immune-mediated adrenal insufficiency** | **1 (0.7)** |
| Adrenal insufficiency | 1 (0.7) |
| **Immune-mediated hypophysitis** | **1 (0.7)** |
| Hypophysitis | 1 (0.7) |
| **Immune-mediated myocarditis** | **1 (0.7)** |
| Myocarditis | 1 (0.7) |
| **Immune-mediated pancreatitis** | **1 (0.7)** |
| Amylase increased | 1 (0.7) |

Data are n (%) unless otherwise specified.

AESI, adverse event of special interest; ALT, alanine aminotransferase; AST, aspartate aminotransferase; GGT, gamma-glutamyl transferase.
